# Mindfulness-Based Cognitive Therapy for Preventing Relapse in Recurrent Depression: A Randomized Dismantling Trial

**DOI:** 10.1037/a0035036

**Published:** 2013-12-02

**Authors:** J. Mark G. Williams, Catherine Crane, Thorsten Barnhofer, Kate Brennan, Danielle S. Duggan, Melanie J. V. Fennell, Ann Hackmann, Adele Krusche, Kate Muse, Isabelle Rudolf Von Rohr, Dhruvi Shah, Rebecca S. Crane, Catrin Eames, Mariel Jones, Sholto Radford, Sarah Silverton, Yongzhong Sun, Elaine Weatherley-Jones, Christopher J. Whitaker, Daphne Russell, Ian T. Russell

**Affiliations:** 1 Department of Psychiatry, University of Oxford, Oxford, United Kingdom; 2 Centre for Mindfulness Research and Practice, Bangor University, Bangor, United Kingdom; 3 North Wales Organisation for Randomised Trials in Health (NWORTH), Bangor University, Bangor, United Kingdom; 4 College of Medicine, Swansea University, Swansea, United Kingdom

**Keywords:** mindfulness-based cognitive therapy, major depression, relapse prevention, suicidality, childhood trauma

## Abstract

***Objective:*** We compared mindfulness-based cognitive therapy (MBCT) with both cognitive psychological education (CPE) and treatment as usual (TAU) in preventing relapse to major depressive disorder (MDD) in people currently in remission following at least 3 previous episodes. ***Method:*** A randomized controlled trial in which 274 participants were allocated in the ratio 2:2:1 to MBCT plus TAU, CPE plus TAU, and TAU alone, and data were analyzed for the 255 (93%; MBCT = 99, CPE = 103, TAU = 53) retained to follow-up. MBCT was delivered in accordance with its published manual, modified to address suicidal cognitions; CPE was modeled on MBCT, but without training in meditation. Both treatments were delivered through 8 weekly classes. ***Results:*** Allocated treatment had no significant effect on risk of relapse to MDD over 12 months follow-up, hazard ratio for MBCT vs. CPE = 0.88, 95% CI [0.58, 1.35]; for MBCT vs. TAU = 0.69, 95% CI [0.42, 1.12]. However, severity of childhood trauma affected relapse, hazard ratio for increase of 1 standard deviation = 1.26 (95% CI [1.05, 1.50]), and significantly interacted with allocated treatment. Among participants above median severity, the hazard ratio was 0.61, 95% CI [0.34, 1.09], for MBCT vs. CPE, and 0.43, 95% CI [0.22, 0.87], for MBCT vs. TAU. For those below median severity, there were no such differences between treatment groups. ***Conclusion:*** MBCT provided significant protection against relapse for participants with increased vulnerability due to history of childhood trauma, but showed no significant advantage in comparison to an active control treatment and usual care over the whole group of patients with recurrent depression.

Depression is a chronic relapsing condition, with relapse rates of 50%–80% and increased risk of suicide (Judd & Akiskal, 2000; Mueller et al., 1999; Penninx et al., 2011; Solomon et al., 2000). High risk of relapse is associated with a history of adversity and abuse, early onset of first episode, and frequent episodes before the index episode (Burcusa & Iacono, 2007). Previous research has indicated that mindfulness-based cognitive therapy (MBCT) reduces risk of relapse by teaching relapse prevention skills to recurrently depressed patients while in remission. It combines psychological education from cognitive therapy for depression with intensive practice of mindfulness meditation (Segal, Williams, & Teasdale, 2002). Six clinical trials have evaluated the effectiveness of MBCT as prophylaxis (Bondolfi et al., 2010; Godfrin & van Heeringen, 2010; Kuyken et al., 2008; Ma & Teasdale, 2004; Segal et al., 2010; Teasdale et al., 2000), and a meta-analysis revealed that, on average, MBCT reduced risk of relapse in patients with three or more prior episodes by 43% relative to treatment as usual (TAU; Piet & Hougaard, 2011). Hence, the UK National Institute of Health and Clinical Excellence (NICE) includes MBCT in its guidelines for the treatment of recurrent depression (National Institute of Health and Clinical Excellence, 2009).

However, evidence is lacking in two respects. First, no study has yet compared MBCT with an active psychological treatment. This means that we do not know to what extent the beneficial effects of MBCT are attributable to the process of learning mindfulness meditation skills rather than to psychological education or nonspecific factors such as group support, despite the fact that mindfulness meditation is widely assumed to be the component critical to effectiveness. In order to examine these issues, we used in this study a “dismantling” design in which cognitive psychological education (CPE) provided a control treatment offering the same educational process and following the same group format as MBCT, but with no training in meditation. Outcomes for both treatment groups were compared with TAU.

Second, evidence is converging to suggest that MBCT is effective only for those at greater risk of relapse, with little evidence of effectiveness for those who are less vulnerable. The two initial trials of MBCT (Ma & Teasdale, 2004; Teasdale et al., 2000) stratified their samples before randomization by the number of prior episodes of major depressive disorder (MDD). Both trials revealed that, although MBCT reduced risk of recurrence in patients with three or more prior episodes, it *increased* risk of recurrence for those with only two prior episodes (Piet & Hougaard, 2011). Furthermore, a recent Canadian trial by Segal et al. (2010) revealed no reduction for patients in stable remission following pharmacotherapy, assigned to either continued antidepressant medication or MBCT, compared with placebo. However, in patients with unstable remission (Nierenberg et al., 2010), MBCT and antidepressants reduced rate of recurrence to 28% and 27%, respectively, compared with a rate of recurrence of 71% in the placebo group.

These studies suggest that MBCT is more effective for more vulnerable patients: those with a greater number of prior episodes of depression or persisting residual symptoms. However, the characterization of patients by number of prior episodes masks other important differences that may be related to the efficacy of MBCT in reducing relapse. For example, the two early trials (Ma & Teasdale, 2004; Teasdale et al., 2000) revealed in exploratory analyses that patients with three or more episodes had an *earlier age of first onset* of MDD, and significantly *more adversity in childhood and adolescence* (more abuse and more indifference from parents), whereas those with only two prior episodes had later onset and did not differ in reported parenting style from a “never depressed” control group. These findings suggest that the differential efficacy of MBCT in people with two versus three or more episodes of depression reflects differences between subpopulations that may differ on a range of clinical risk factors (Ma & Teasdale, 2004). We therefore tested whether each variable previously associated with better outcome also moderated efficacy of MBCT in the current trial. We also stratified the sample for history of suicidality to examine whether MBCT had differential effectiveness in those with this risk factor. This article focuses on the main outcome of the trial, time to relapse to major depression—the standardized outcome reported in all previous trials of MBCT for depression and the basis of the meta-analysis (Piet & Hougaard, 2011).

## Method

### Participants

We recruited participants through referrals from primary care and mental health clinics in Oxford, England, and Bangor, North Wales, and advertisements in the community. We assessed eligibility through the Structured Clinical Interview for *DSM–IV*, the *Diagnostic and Statistical Manual of Mental Disorders* (SCID; First, Spitzer, Gibbon, & Williams, 2002). Inclusion criteria at baseline assessment were (a) age between 18 and 70 years; (b) history of at least three episodes of major depression meeting *DSM–IV*, text revision (*DSM–IV–TR*) criteria (American Psychiatric Association, 2000), of which two must have occurred within the last 5 years, and one within the last 2 years; (c) remission for the previous 8 weeks (with potential trial participants deemed *not* to be in recovery or remission, and hence *ineligible*, if they reported that at least 1 week during the previous 8 they experienced *either* a core symptom of depression (depressed mood, anhedonia) *or* suicidal feelings and at least one other symptom of depression, which together were not attributable to bereavement, substances, or medical condition, but were impairing functioning); and (d) informed consent from participants and their primary care physicians.

Exclusion criteria were (a) history of schizophrenia, schizoaffective disorder, bipolar disorder, current abuse of alcohol or other substances, organic mental disorder, pervasive developmental delay, primary diagnosis of obsessive-compulsive disorder or eating disorder, or regular nonsuicidal self-injury; (b) positive continuing response to cognitive behavior therapy (CBT), that is, no relapse to depression since treatment with CBT, due to the known effects of CBT in reducing risk of relapse; (c) current psychotherapy or counseling more than once a month; (d) regular meditation practice (meditating more than once per month); or (e) inability to complete research assessments through difficulty with English, visual impairment, or cognitive difficulties.

### Randomization

We aimed to recruit 300 participants and randomly allocate them between groups in the ratio 120 MBCT + TAU: 120 CPE + TAU: 60 TAU alone. This would yield 99% power for survival analysis with 5% significance level to detect the predicted difference of 31% in recurrence between MBCT and TAU, and 80% power to detect the predicted difference of 18% between MBCT and CPE (Williams et al., 2012). Reanalysis of the two original trials of MBCT (Ma & Teasdale, 2004; Teasdale et al., 2000) yielded negative estimates of intraclass correlation for time to relapse (Williams, Russell, & Russell, 2008). Thus, there was no evidence of intraclass dependency or loss of power through clustering. Hence, we were able to treat participants as independent for the purpose of power calculations. Randomization was by e-mail to the North Wales Organisation for Randomised Trials in Health, which used dynamic allocation (Russell, Hoare, Whitaker, Whitaker, & Russell, 2011) to stratify by two variables in addition to site and recruitment cohort: antidepressant medication in last 7 days and history of suicidality. Participants provided written consent before assessment of eligibility and renewed that consent before randomization. Participants were informed of the outcome of randomization by letter and also by e-mail or telephone if desired. Once they had been notified of their allocation, they were invited to schedule a preclass interview (in the case of those allocated to MBCT or CPE) or a TAU interview for those in the TAU arm.

Assessors derived information for stratification about past suicidality from the suicidality questions of the SCID, Item 20 of the Beck Scale for Suicidal Ideation (Beck & Steer, 1993) and the Suicide Attempt and Self-Injury Interview (Linehan, Comtois, Brown, Heard, & Wagner, 2006). The main variables derived from the SCID to characterize past depression were age of onset; number of prior episodes; and occurrence of chronic depression as defined by the *DSM–IV–TR*, namely, major depression that lasted for at least 2 years. From the SCID, we also derived binary measures of current or past anxiety disorder and of current or past substance dependence or abuse. We asked participants to report any antidepressant medication they had taken during the previous week. We assessed residual depressive symptoms at trial entry by the Hamilton Rating Scale for Depression (HAMD; Hamilton, 1960), using the 17-item version of this interviewer-rated scale. Internal consistency for the HAMD in our study was α = .73. Additionally, participants provided self-reports on the Beck Depression Inventory–II (BDI-II), a well-established questionnaire measure of depressive symptomatology that contains 21 groups of statements, referring to the presence of symptoms over the preceding 2 weeks (Beck, Steer, & Brown, 1996). Internal consistency for the BDI-II was α = .90. Early adversity and abuse were assessed using the Childhood Trauma Questionnaire (CTQ; Bernstein & Fink, 1997), an established self-report measure containing 28 items, validated against interview measures of childhood trauma and maltreatment (Bernstein & Fink, 1997; Bernstein et al., 1994). Internal consistency of a sumscore combining scores from five subscales on emotional, physical, and sexual abuse, and emotional and physical neglect showed an internal consistency of α = .94. Higher scores indicate greater severity for all three measures.

### Research Governance

The Oxfordshire Research Ethics Committee C and the North Wales Research Ethics Committee approved the trial in July 2008, and several subsequent operational changes. Thereafter, an independent Trial Steering Committee and Data Monitoring Subcommittee oversaw the trial.

### Interventions

Six cohorts received treatment between January 2009 and December 2010. We encouraged participants in all three groups to continue current medication and attend their mental health practitioners or other services as usual during the trial (TAU). We informed participants’ general practitioners of their patients’ allocated treatment, the dates of treatment sessions (if applicable), and the point at which patients ended their participation in the trial.

#### MBCT

MBCT is a manualized group skills training program (Segal et al., 2002) that integrates psychological educational aspects of CBT for depression with meditation components of mindfulness-based stress reduction developed by Kabat-Zinn (1990). It stems from experimental research showing that relapse is more likely when, in periods of low mood, patterns of negative thoughts and feelings associated with previous episodes of depression recur (Lau, Segal, & Williams, 2004). The program teaches skills that enable participants to disengage from these habitual dysfunctional cognitive routines and thus reduce the risk of relapse into depression. In this study, MBCT comprised an individual preclass interview followed by eight weekly 2-hr classes, including training in meditation skills such as sustained attentional focus on the body and breath and adopting a decentered view of thoughts as passing mental events. The program followed the original MBCT manual (Segal et al., 2002) except for greater emphasis on patterns of thoughts and feelings that might be associated with suicidal planning, factors that maintain and exacerbate such patterns, and preparation of explicit action plans for suicidal crises. Participants were also invited to follow-up classes taking place 6 weeks and 6 months posttreatment, respectively. Each follow-up class lasted for 2 hr and included meditation, discussion of discoveries and difficulties since the course ended, and how these were being dealt with by participants.

#### CPE

The CPE program, developed for this trial, comprised all elements of the MBCT program *except* the experiential cultivation of mindfulness through meditation practice and followed the same format of eight weekly 2-hr classes (i.e., matched for time with MBCT), with follow-up classes at 6 weeks and 6 months. Thus, participants learned about the psychological processes of relapse and were encouraged to apply what they had learned outside the sessions, to help prevent relapse. They also learned how to recognize the warning signs of depression and the importance of disengaging from unhelpful processes such as rumination and experiential avoidance. CPE educated them about these processes through interactive practical exercises and group discussions. CPE participants completed all MBCT homework that involved no meditation practice (pleasant and unpleasant events calendar, automatic thoughts worksheets, relapse signature warning signs worksheets, and how to plan for times of high relapse vulnerability—around 2 hr of homework in all). However, the dismantling design of the study meant that we intentionally did not match the CPE group for amount of homework with MBCT, because CPE did not include home meditation practice, and addition of a substitute homework (often preferred in other pragmatic designs) would have made our key scientific question unanswerable (see the Discussion section).

CPE participants were offered MBCT once data collection was completed. Further details of the interventions are provided at http://oxfordmindfulness.org/science/projects/staying-well.

#### Treatment engagement interview

Data from a pilot trial of MBCT for patients with a history of suicidality in the context of a unipolar or bipolar mood disorder (Williams et al., 2008) identified high rates of attrition from treatment, with further analysis indicating that attrition often occurred early in the treatment process and in individuals who showed high levels of cognitive reactivity to changes in mood and who were more prone to ruminative brooding (Crane & Williams, 2010). In order to enhance treatment engagement in the current trial, considerable attention was paid to the preclass interview participants received. Therapists received a summary of the clinical history information derived from the baseline assessment, including details of any prior suicidal ideation or suicidal behavior. During the interview, therapists and participants reviewed the participant’s history of depression and other problems and jointly developed a formulation of how MBCT or CPE would address these. Participants with high levels of brooding were flagged, and therapists made a particular point of discussing difficulties that might arise during treatment, at times when it seemed that no progress was being made in dealing with negative thoughts and feelings. Participants were welcome to contact the therapist between sessions if they were experiencing difficulties and therapists actively followed up participants who did not attend treatment sessions, sending out the relevant handouts and other materials by mail and attempting to schedule a telephone call to cover information that had been missed.

#### TAU

TAU began with an interview with a trial therapist or trained recruiter. Given that all participants were currently in remission, there was no attempt made to specify what TAU should be. Nevertheless, the therapist stressed the importance of the participant seeking treatment as needed from their usual health care provider throughout their period in the trial, and records were kept of the treatment sought at each assessment point. These data indicated that 21% of patients in TAU received one or more new antidepressant prescriptions during the follow-up phase, in comparison to 18% of participants in MBCT and 13% of those in CPE. Similarly, 11% of those in TAU saw a psychiatrist or community psychiatric nurse regarding their depression at least once during the follow-up phase in comparison to 10% of those allocated to MBCT and 9% of those allocated to CPE. Finally, 21% of those allocated to TAU saw a counselor, psychologist, or psychotherapist at least once during the follow-up phase, compared with 18% of those allocated to MBCT and 12% of those allocated to CPE. There were no significant differences between groups in pharmacological or psychiatric/psychological treatment received. All participants were offered their treatment of choice once data collection was over, regardless of whether they were in the MBCT, CPE, or TAU arm initially.

#### Therapist training and fidelity

Four therapists delivered both MBCT and CPE, each leading six classes, and alternating between the two therapies. All therapists were instructors in MBCT with at least 3 years’ experience; all contributed to developing CPE and piloting it at each of the two sites. During the trial, JMGW ensured treatment fidelity by monitoring treatment adherence and competence for both MBCT and CPE through weekly video conferences and the viewing of video recordings of classes. Treatment fidelity ratings on the Mindfulness-Based Interventions–Teaching Assessment Criteria Scale (Crane et al., 2012, 2013; see also http://mindfulnessteachersuk.org.uk/pdf/MBI-TACJune2012.pdf), which assesses therapist adherence to the MBCT protocol and competence in its delivery, was 5.6 out of 6 (with modal scores in the highest category: “Advanced”) across the four therapists.

#### Treatment credibility

Participants allocated to MBCT or CPE rated the credibility of their allocated treatment at the start of Session 2 on three scales from 0 to 10. Though these scales slightly favored CPE, there were no significant differences between MBCT and CPE groups in how sensible the treatment seemed (mean difference = −0.60; 95% CI [−1.25, 0.06]); confidence in treatment’s capacity to prevent future depression (mean difference = −0.38; 95% CI [−1.00, 0.24]); or confidence in recommending treatment to a friend with similar problems (mean difference = −0.40; 95% CI [−1.07, 0.28]).

### Assessment of Primary Outcomes

We assessed participants from September 2008 until December 2011 using fully trained assessors blind to treatment allocation—before randomization; immediately after treatment (or at the equivalent point for the TAU group); and then at 3, 6, 9, and 12 months after treatment or equivalent. We strove to maintain blinding: For example, we assessed participants in different buildings from those in which treatment took place and checked assessor blindness at the end of every assessment session. On the rare occasions on which an assessor became unblinded due to overt disclosure by a participant, we used an alternative assessor for all subsequent assessments.

The primary outcome was *time until relapse to major depression*, defined as meeting relevant SCID criteria for at least 2 weeks since the previous assessment. Participants were asked to date the onset of their episode as accurately as possible. Where they could not give a precise date of onset, we used an algorithm to approximate the date of onset for derivation of “a days to relapse” variable.^1^

We recorded all SCIDs and recruited two independent psychiatrists to reassess a sample of 91 follow-up interviews. Psychiatrists received only the audio-recording of the clinical interview. Cases for independent review were selected by a researcher blind to all participant details, who had not listened to the audio-recordings, with the aim of including approximately two relapses per assessor, per cohort, per assessment wave (posttreatment, 3, 6, 9, 12 months). Once each relapse interview had been identified, the next available case within a cohort and without a relapse at the assessment in question was selected as a control. Interrater reliability between the original assessor and an independent psychiatrist was 0.74, 95% CI [0.60, 0.87], with 87% agreement on whether relapse had occurred.

We monitored serious adverse events (SAEs) and reported these to the trial Data Monitoring Subcommittee and to the National Research Ethics Service if potentially arising from a trial intervention.

### Statistical Analysis

Data were analyzed by treatment allocated. In the current trial, only 7% of participants dropped out before providing any follow-up data, and analysis of baseline characteristics revealed no significant differences between those who dropped out before providing follow-up data and the remainder of the sample, other than a small difference in age. We used Cox proportional hazards regression models (Cox, 1972; Singer & Willett, 2003), which censored cases at relapse, or in the case of those who were nonrelapsed and dropped out before 12 months, at the last date of follow-up. In accordance with the agreed-upon analysis plan (Williams et al., 2010, 2012), we first tested the univariate effects of stratification variables and potential moderators, and their interactions with treatment group, on time to relapse by the Cox proportional hazards regression model. We then used a forward stepwise approach to derive a parsimonious model predicting risk of relapse from treatment allocated and stratifying and moderator variables, including interactions between group and other variables. This model enabled us to examine potential interactions between predictor variables and to identify the strongest predictors among a set of variables that was likely to be correlated. The research team remained blind to which groups received MBCT and CPE until the Trial Steering Committee had reviewed the primary analyses. The funder played no role in study design or conduct, or the decision to submit this article for publication.

## Results

**Figure 1 fig1:**
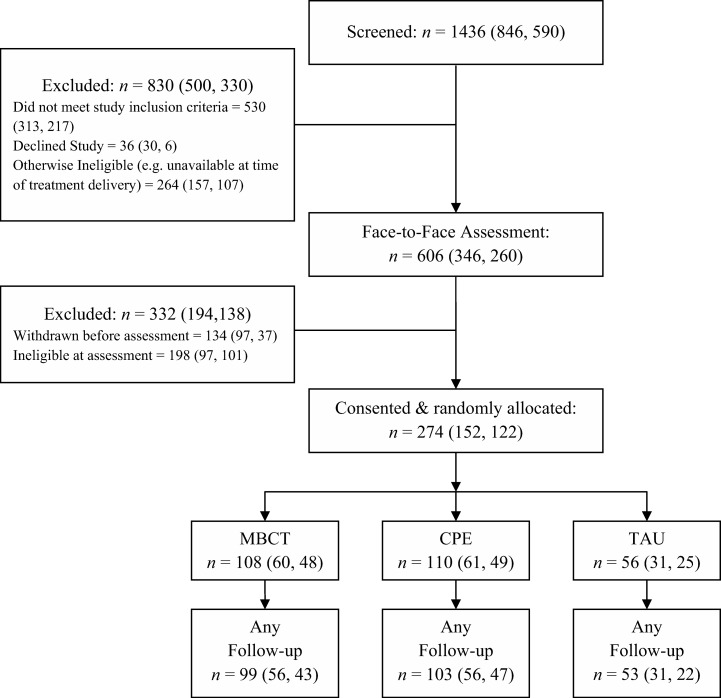
Flow of participants through the study. Numbers in parentheses denote participants at Oxford, first, and Bangor, second. MBCT = mindfulness-based cognitive therapy; CPE = cognitive psychological education; TAU = treatment as usual.

We enrolled and randomized 274 patients between September 2008 and October 2010 (see Figure 1). Nineteen (7%) left the trial before any follow-up data could be collected: nine from MBCT, seven from CPE, and three from TAU. Of these, two explicitly withdrew from the trial, one moved away, and the remaining 16 repeatedly failed to attend scheduled follow-up appointments. Thus, follow-up data were available for 255 participants: 99 receiving MBCT, 103 CPE, and 53 TAU. The mean time from randomization to final follow-up did not differ significantly between groups: 467 days for MBCT; 446 days for CPE; and 462 days for TAU, *F*(2, 252) = 1.25, *p* = .29.[Fig-anchor fig1]

Both treatment groups attended a median of seven of the eight treatment sessions; 89 (90%) of MBCT participants completed four or more sessions, compared with 97 (94%) for CPE. Participants in MBCT completed a daily homework diary in which they recorded whether or not they had completed a formal meditation practice, alongside specific informal practices, which differed from week to week. Where homework data were missing, we made a conservative assumption that no homework had been completed. Participants completed a formal meditation practice on an average of 3.36 days per week (*SD* = 1.77; range = 0–6.43). There was no significant difference in amount of practice between participants at the two study sites, with limited evidence of decline in practice across treatment weeks (Week 1: *M* = 3.81, *SD* = 2.22; Week 7: *M* = 3.00, *SD* = 2.60).

Participants reported 15 SAEs to the research team, five arising from MBCT and 10 from CPE. We adjudged only one to be a “serious adverse reaction” potentially arising from a trial treatment—an episode of serious suicidal ideation following discussion of different coping responses to low mood in CPE. The rest were overnight admissions: 13 for physical health problems and one following an overdose during follow-up in a patient who had received MBCT. One participant died from an unrelated medical condition after partially withdrawing from trial follow-up due to illness.

### Demographic and Clinical Characteristics

The 274 participants had a mean age of 43 years (*SD* = 12 years; range = 18–68) at entry to the study; 198 (72%) were female, with 13 (5%) describing themselves as non-Caucasian. Table 1 shows the baseline characteristics of those who provided follow-up data by treatment group. The 19 participants lost to follow-up were significantly younger than those who provided follow-up data, by 5.6 years (95% CI [1.5, 9.7]). There were no other significant differences between the groups.[Table-anchor tbl1]

### Relapse to Major Depression

The raw relapse rates for the 255 participants (93%) with follow-up data were 46% in the MBCT group, 50% in the CPE group, and 53% in the TAU group. Table 2 shows the main effects of treatment group and the stratification and moderator variables, and their interactions with treatment group. Treatment group had no significant main effect on risk of relapse (*p* = .56). There were significant univariate effects of site, residual symptoms (the HAMD), and childhood trauma (the CTQ). Participants in the Bangor site were more likely to relapse during the study period (hazard ratio = 1.56, 95% CI [1.10, 2.21]), as were those with higher HAMD or childhood trauma scores. The hazard ratios for an increase of one standard deviation at baseline were 3.54 for HAMD (OR = 1.26, 95% CI [1.07, 1.48]) and 17.79 for childhood trauma (OR = 1.28, 95% CI [1.09, 1.52]).[Table-anchor tbl2]

We used a forward stepwise approach to derive the most parsimonious predictive model of time until relapse from treatment group and all stratifying and moderator variables (see Table 3). The final model included treatment group (not significant but essential in the presence of interaction); site (hazard ratio now 1.43, 95% CI [0.98, 2.09]—no longer significant, but also needed to characterize interaction); childhood trauma (hazard ratio for increase of 1 *SD* = 1.26, 95% CI [1.05, 1.50]); HAMD (hazard ratio for increase of 1 *SD* = 1.23, 95% CI [1.03, 1.46]); a Childhood Trauma × Treatment Group interaction (hazard ratios for increase of 1 *SD* = 0.42, 95% CI [0.26, 0.69] for MBCT vs. TAU; and 0.51, 95% CI [0.33, 0.79] for MBCT vs. CPE); and a Site × Treatment Group interaction (hazard ratio = 1.93, 95% CI [0.74, 5.02], for MBCT vs. TAU; and 2.76, 95% CI [1.23, 6.21] for MBCT vs. CPE). This Site × Treatment Group interaction, which emerged only in stepwise modeling, resulted from a greater reduction in risk of relapse in those allocated to MBCT in Oxford than in Bangor.[Table-anchor tbl3]

The significant Childhood Trauma × Treatment Group interaction resulted from differences between MBCT and both other trial arms in the dependence of risk of relapse on childhood trauma score. To characterize this interaction, we conducted further exploratory analyses, dichotomizing the sample at the median childhood trauma score (*Mdn* = 39.00) into high (*n* = 126) or low (*n* = 129) scorers and examining the effect of treatment group on the hazard ratio of relapse for each subsample. These analyses have lower power than the full model of Table 3, but illustrate the effect of the interaction in that model.

**Figure 2 fig2:**
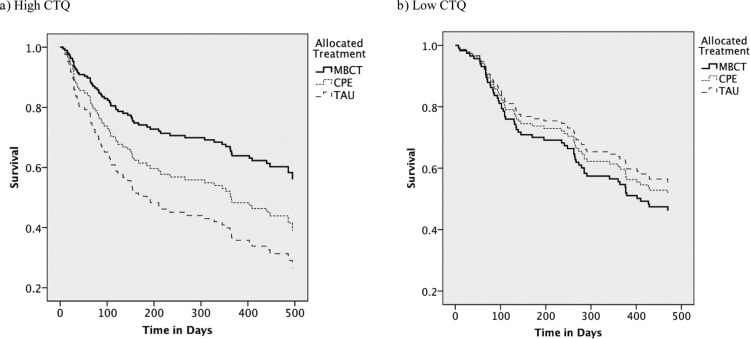
Proportions of patients who survived without relapse during follow-up in those with high (a: *n* = 126) and low (b: *n* = 129) Childhood Trauma Questionnaire (CTQ) scores. MBCT = mindfulness-based cognitive therapy plus TAU; CPE = cognitive psychological education plus TAU; TAU = treatment as usual.

For high childhood trauma scorers, raw rates of relapse were 41%, 54%, and 65% for MBCT, CPE, and TAU, respectively. Cox regression showed that treatment with MBCT yielded a significant hazard ratio for subsequent relapse of 0.43 (95% CI [0.22, 0.87]) relative to TAU (see Figure 2a). In contrast, MBCT yielded a nonsignificant hazard ratio of 0.61 (95% CI [0.34, 1.09]) relative to CPE. Table 4 also shows that CPE did not differ significantly from TAU (hazard ratio = 0.72, 95% CI [0.39, 1.32]).[Fig-anchor fig2][Table-anchor tbl4]

For low childhood trauma scorers, the rates of relapse were 51%, 45%, and 43% for MBCT, CPE, and TAU, respectively (see Figure 2b). None of the pairwise Cox regressions were significant in this subgroup (see Table 4).

## Discussion

### Principal Findings

Previous studies (Bondolfi et al., 2010; Godfrin & van Heeringen, 2010; Kuyken et al., 2008; Ma & Teasdale, 2004; Segal et al., 2010; Teasdale et al., 2000) have shown that MBCT protects vulnerable patients from depression for at least 12 months after treatment, yielding a preventative effect similar to that of continued antidepressant medication (Piet & Hougaard, 2011). In contrast, this study revealed no significant general risk reduction in those allocated to MBCT compared with TAU or CPE.

However, analyses did show that for those with more history of childhood trauma, MBCT made a significant difference: The hazard ratio of relapse for patients receiving MBCT was 0.43 relative to those receiving TAU alone, though in this model, the contrast between MBCT and CPE fell short of significance (see below). For those with less or no history of childhood trauma, MBCT did not reduce risk of relapse compared with TAU. In the more powerful full model, the severity of experience of childhood trauma significantly moderated both the contrasts between MBCT and CPE, and between MBCT and TAU. This suggests that, overall, MBCT is more effective in preventing relapse to major depression than both CPE and TAU when severity of childhood trauma is taken into account. However, these results should be considered in the context of the strengths and limitations of the trial.

The strength of this study is that it is the largest trial of MBCT versus TAU to date, and the first in which MBCT has been compared with an active control treatment. Furthermore, we had very high rates of compliance with treatment, with more than 90% of participants allocated to both active treatments completing four or more sessions, which previous trials adjudged an adequate minimum dose. The trial also had low rates of dropout, with 93% of participants providing follow-up data. We maximized external validity by including patients who had achieved remission by antidepressant medication, counseling, or psychotherapy other than CBT, or no treatment at all, and included patients who were on antidepressants at entry to the trial. This suggests that the results reported here are generalizable to routine clinical practice. As the vast majority (95%) of the sample was Caucasian, however, we cannot be sure that these findings generalize to other ethnic groups.

By design, we did not balance the amount of homework between the two active treatments. Because there is no meditation practice in CPE, the volume of homework was less. Thus, we cannot be sure that the superiority of MBCT over CPE and TAU in participants reporting a history of childhood trauma was due to the inclusion of mindfulness meditation in MBCT but not in CPE, rather than the requirement for regular treatment-related activity outside sessions. The fact that the effects of CPE were intermediate between MBCT and TAU suggests that some of the effect of MBCT may be explained by the psychological education and group support provided by both MBCT and CPE interventions. For participants with a history of childhood trauma, although a significant difference emerged between MBCT and CPE as part of the significant interaction for the whole model, loss of power when the group was divided for exploratory analysis meant that the pairwise comparison between MBCT and CPE fell short of significance at this point, despite a relatively large hazard ratio of 0.61 (a reduction in hazard of 39%). To examine whether the amount of homework in MBCT affects the outcome will require a trial that randomly allocates patients to treatment arms with different homework schedules.

Two other aspects of the results deserve comment. First, we found no main effect of antidepressant medication use at entry on relapse. At first sight, this seems different from previous trials that evidence that continued use of antidepressant medications prevent relapse. However, note that these trials focus on antidepressant medication as a treatment arm, and patients are randomly allocated to maintenance medication as part of a design to examine their efficacy (e.g., Segal et al., 2010). In the current trial, we stratified for this variable only to check whether antidepressant medications interacted with treatment allocated and did not examine antidepressant medication use further because such ongoing use is multidetermined, was neither controlled nor randomly assigned, and might therefore lead to misleading results. The fact that antidepressant medication use at entry to the trial did not affect outcome does not imply that antidepressant medications are ineffective, because those who were taking antidepressant medications may have more severe clinical status at entry to the trial and more likelihood of returning for treatment. Second, we found evidence of a Site × Treatment effect, due to a greater effectiveness of MBCT in Oxford. We checked the therapist fidelity data and could find no hint of a discrepancy in therapist competence that might explain the site difference, nor did the sites differ in perceptions of treatment credibility or homework compliance. We then looked for population differences that might have accounted for it. Participants in Oxford were more likely to be single and employed and had more years of full-time education than participants in Bangor. However, none of these variables interacted with treatment group to predict relapse. It remains possible that subtle but unmeasured sociocultural differences in the two populations from which the samples were drawn explains this effect (e.g., therapists at Bangor reported a repeated tendency for participants in their groups to “hear” an invitation during a meditation practice as if it was an instruction they had to obey, and thereby to see the meditation as a success–failure issue), and we suggest that future trials examine such issues systematically.

Finally we did not standardize the usual care that patients received. All patients were encouraged to continue with existing pharmacotherapy and seek further treatment as needed throughout the trial. We stratified participants by use of antidepressants at entry to the trial and excluded those who were receiving counseling or psychotherapy more than once a month at entry. Because all patients were in remission at entry to the trial, the issue of usual care is less critical than for an acute treatment trial. However, an examination of care received in each group during the follow-up phase identified no significant differences between treatment arms, and relatively low levels of engagement in alternative treatment across all three groups.

Although the overall absence of a difference between MBCT and TAU in preventing relapse to depression is in contrast to the results of the most recent meta-analysis of MBCT as prophylaxis in major depression (Piet & Hougaard, 2011), the finding that benefits were observed for more vulnerable participants is consistent with a number of earlier studies. Indeed, earlier studies have revealed that MBCT yielded additional benefit over TAU or placebo only for those who were vulnerable to relapse, identified variously across studies as those with only three or more prior episodes, those with earlier onset (Ma & Teasdale, 2004; Teasdale et al., 2000), those with a history of abuse or adversity (Ma & Teasdale, 2004), or those failing to achieve stable remission (Segal et al., 2010). The findings of the present trial that the more vulnerable patients were those with a history of childhood trauma and that it was this group that benefited from MBCT is consistent with these results. However, all these variables tend to be correlated with one another, and we do not assume that this index of vulnerability will necessarily always be the one that predicts better outcome. The present sample included a greater proportion of patients with a history of suicidal ideation or behavior than previous studies, and it is known that childhood trauma is a particularly salient risk factor in this population. The balance of evidence to date suggests that the variable that most strongly predicts vulnerability to relapse within any given clinical population may also be the one most likely to interact with treatment condition and predict greater benefit from prophylactic treatment.

Although the findings suggest that, when severity of childhood trauma is taken into account, MBCT is superior to both an alternative psychological treatment and TAU in preventing recurrence of depression over 12 months, there were no general differences in outcome according to treatment allocated in the present trial. The crude rate of relapse in the TAU arm of the present trial, at 53%, was low compared with earlier studies that also looked at patients with three or more prior episodes (e.g., 66% in the first trial of MBCT, Teasdale et al., 2000, and 78% in its replication, Ma & Teasdale, 2004). This improved outcome for people receiving usual care in the present trial may reflect improved management of patients with major depression in the 13 years since the first trial of MBCT. It is also possible that given NICE guidelines in the UK health system, TAU may contain more empirically supported interventions than previous research or current practice in other studies. This potentially makes even the TAU control very strong, and might be contributing to the lack of findings.

Additionally, because our participants had a higher frequency of prior suicidality than has been typical in previous trials, the primary care physicians may have been more vigilant in the TAU condition, which may partially explain the lack of a main effect of treatment on outcome, similar to that suggested by the authors of one previous trial of MBCT (Bondolfi et al., 2010).

The overall pattern of data from this and previous trials in which the more vulnerable patients appear to do well with MBCT is different from that observed with psychological treatments for acute depression, such as cognitive therapy. For acute treatments, those with better premorbid history show a better response to treatment, whereas those with early age of onset, greater number of prior episodes, and a history of trauma tend to do less well (Harkness, Bagby, & Kennedy, 2012; Nanni, Uher, & Danese, 2012). It is possible that this may not be a difference between MBCT and these acute treatments themselves. Instead, the relevant difference may be between *any* psychosocial approach when used for acute treatment and when used prophylactically. For example, two studies using cognitive therapy as prophylaxis revealed better outcomes for those who were more vulnerable (Bockting et al., 2005; Jarrett et al., 2001). We suggest that prophylactic treatment may have greater benefits for more vulnerable patients in remission because, despite the absence of acute symptoms, their habitual dysfunctional thought patterns react to slight changes in mood, and can thus become the focus of the treatment (Hollon, Thase, & Markowitz, 2002; Lau et al., 2004; Williams, Barnhofer, Crane, & Beck, 2005). Once in remission, those who are less vulnerable may have limited access to mood-related reactivation of dysfunctional content, so neither cognitive therapy nor MBCT adds therapeutic effect. However, it remains true that MBCT may have been particularly beneficial for those with a history of childhood trauma because certain elements of the approach (e.g., acceptance, self-compassion, and decentering) are well matched for addressing the factors that increase risk in these patients, particularly in the adapted form used in the present trial. Indeed, MBCT may facilitate emotional processing by encouraging participants to remain in contact with painful material rather than avoiding it or becoming entangled in rumination about it. However, further research, comparing CT and MBCT as prophylactic treatments, would be necessary to test these specific hypotheses.

In short, our findings add to the growing body of evidence that psychological interventions, delivered during remission, may have particular beneficial effects in preventing future episodes of major depression, but may be especially relevant for those at highest risk of relapse. The pattern of data that emerges from all existing trials suggests that future research should now give particular attention to characterizing indices of vulnerability so that the particular treatment characteristics that might address them can be clearly identified.

## Figures and Tables

**Table 1 tbl1:** Descriptive Statistics for Participants With and Without Follow-Up Data and for Those With Follow-Up Data by Allocated Treatment

Variable	Comparison between				
	Participants assessed at outset			Participants followed-up, by allocated treatment	
	With follow-up	Without follow-up	MBCT	CPE	TAU
	(*n* < 255)	(*n* < 19)	(*n* < 99)	(*n* < 103)	(*n* < 53)
Female, *n* (%)	183 (72)	15 (79)	70 (71)	76 (74)	37 (70)
Age at enrolment, *M* (*SD*)^a^	43.82 (12.17)	38.21 (8.05)	43.99 (11.55)	43.86 (12.92)	43.43 (12.03)
Ethnicity (Caucasian), *n* (%)	244 (96)	17 (89)	95 (96)	97 (94)	52 (98)
Employed at enrollment, *n* (%)	159 (62)	13 (68)	68 (69)	57 (55)	34 (64)
Age of onset of major depression, *M* (*SD*)^b^	21.26 (10.93)	16.84 (6.44)	20.64 (9.62)	21.28 (11.78)	22.34 (11.61)
Number of previous episodes major depression (5 or more), *n* (%)	196 (77)	16 (84)	71 (72)	85 (83)	40 (76)
Past suicidality (ideation/behavior), *n* (%)	203 (80)	18 (95)	80 (81)	82 (80)	41 (77)
SCID diagnosis of anxiety disorder, *n* (%)^c,e,f^	102 (40)	6 (30)	34 (35)	44 (44)	24 (45)
SCID diagnosis of alcohol/substance abuse/dependence, *n* (%)^d,e^	36 (15)	2 (11)	17 (18)	12 (13)	7 (14)
Antidepressant use at enrollment, *n* (%)	114 (45)	6 (32)	44 (44)	47 (46)	23 (43)
Hamilton Rating Scale for Depression at enrollment, *M* (*SD*)	3.20 (3.54)	3.84 (4.11)	3.17 (3.61)	3.55 (3.50)	2.57 (3.47)
Childhood Trauma Questionnaire score, *M* (*SD*)	43.81 (17.79)	44.34 (13.99)	41.78 (15.62)	46.61 (18.84)	42.15 (19.05)
Beck Depression Inventory, *M* (*SD*)	8.04 (8.11)	11.12 (10.52)	7.72 (6·68)	8.86 (9.27)	7.05 (6.94)
*Note*. MBCT = mindfulness-based cognitive therapy; CPE = cognitive psychological education; TAU = treatment as usual; SCID = Structured Clinical Interview for *DSM–IV*.					
^a^ Participants lost to follow-up were significantly younger than those who provided follow-up data (mean difference = −5.61, 95% CI [−9.73, 1.49]; *p* = .010). ^b^ Data available for 273 participants (missing: one allocated to MBCT). ^c^ Data available for 269 participants (missing: one lost to follow-up; two from MBCT and two from CPE among those with follow-up). ^d^ Data available for 262 participants (missing: two from MBCT, seven from CPE, and three from TAU, all among those with follow-up). ^e^ Percentages of those with available data. ^f^ Among those with follow-up data, four individuals met criteria for current posttraumatic stress disorder (PTSD), six reported symptoms of PTSD in partial remission, and 24 reported a history of PTSD (in full remission/prior history).					

**Table 2 tbl2:** Cox Regressions for Time to Relapse: Individual Stratification and Moderator Variables and Their Interactions With Treatment Group

Variable	Hazard ratio (HR)	95% CI for HR	Change in max. log likelihood	*df*	*p*
Group			1.17	2	.56
MBCT vs. CPE	0.83	[0.56, 1.24]			
MBCT vs. TAU	0.79	[0.49, 1.27]			
Stratification variables					
Suicidality	1.11	[0.86, 1.43]	0.71	1	.40
Group × Suicidality			3.83	2	.15
Antidepressant use at baseline	1.17	[0.82, 1.66]	0.81	1	.37
Group × Antidepressant Use at Baseline			3.46	2	.18
Site (B)	1.55	[1.09, 2.20]	6.05	1	.01
Group × Site			5.73	2	.05
MBCT vs. CPE	2.59	[1.15, 5.81]			
MBCT vs. TAU	2.07	[0.80, 5.37]			
Cohort			8.33	5	.14
Group × Cohort			15.42	10	.12
Moderators					
Beck Depression Inventory (BDI)	1.01	[0.99, 1.03]	1.50	1	.21
Group × BDI			2.15	2	.34
Hamilton Rating Scale for Depression (HAMD)	1.06	[1.02, 1.11]	7.29	1	.01
Group × HAMD			1.76	2	.42
Childhood Trauma Questionnaire (CTQ)	1.01	[1.00, 1.02]	7.63	1	.01
Group × CTQ			11.31	2	.00
MBCT vs. CPE	0.97	[0.94, 0.99]			
MBCT vs. TAU	0.95	[0.93, 0.94]			
Age of onset of major depression (Onset)^a^	0.99	[0.97, 1.01]	0.87	1	.35
Group × Onset			5.05	1	.08
MBCT vs. CPE	0.96	[0.92, 1.00]			
MBCT vs. TAU	0.94	[0.90, 0.99]			
Number of episodes of MDD (episodes)	1.01	[0.98, 1.04]	1.07	1	.30
Group × Episodes			0.78	2	.68
*Note*. CI = confidence interval; max. = maximum; MBCT = mindfulness-based cognitive therapy; CPE = cognitive psychological education; TAU = treatment as usual; B = Bangor.					
^a^ Analysis of 254 participants.					

**Table 3 tbl3:** Cox Regression for Time to Relapse: Multivariate Model Examining Variables Associated With Hazard of Relapse to Major Depression

Variable^a^	*df*	*p*	Max. log likelihood		Change in max. log likelihood
Block 0: Initial log likelihood			1304.26		
Block 1: Group					
1. Group (forced entry)	2	.56	1303.08		1.17
Block 2: Stratification variables					
2. Site	1	.01	1297.04		6.05
Block 3: Modifier variables					
3. CTQ	1	.01	1290.43		6.60
4. HAMD		.02	1285.86		4.57
Block 4: Interactions					
5. CTQ × Group	2	.00	1272.43		13.44
6. Site × Group	2	.04	1266.19		6.24
Final model	9	<.01	1266.19		38.07
Components of final model	Wald	*df*	*p*	Hazard ratio (HR)	95% CI for HR
Group	2.28	2	.32		
MBCT vs. CPE	0.34	1	.56	0.88	[0.57, 1.34]
(MBCT or CPE) vs. TAU	1.98	1	.16	0.73	[0.47, 1.13]
MBCT vs. TAU^b^	2.26	(1)	.13	0.68	[0.42, 1.12]
Site	3.43	1	.06	1.43	[0.97, 2.09]
CTQ	6.42	1	.01	1.01	[1.00, 1.02]
HAMD	5.31	1	.02	1.06	[1.00, 1.11]
CTQ × Group	13.56	2	.00		
CTQ × (MBCT vs. CPE)	9.02	1	.00	0.96	[0.94, 0.98]
CTQ × (MBCT or CPE) vs. TAU	7.24	1	.00	0.97	[0.95, 0.99]
CTQ × (MBCT vs. TAU)^b^	12.46	(1)	<.01	0.95	[0.92, 0.97]
Site (B) × Group	6.12	2	.04		
Site × (MBCT vs. CPE)	6.06	1	.01	2.76	[1.23, 6.21]
Site × (MBCT or CPE) vs. TAU	0.11	1	.74	1.15	[0.49, 2.71]
Site × (MBCT vs. TAU)^b^	1.79	(1)	.18	1.92	[0.73, 5.01]
*Note*. Max. = Maximum; CTQ = Childhood Trauma Questionnaire; HAMD = Hamilton Rating Scale for Depression; CI = confidence interval; MBCT = mindfulness-based cognitive therapy; CPE = cognitive psychological education; TAU = treatment as usual; B = Bangor.					
^a^ Analysis of 255 participants with follow-up data. Other variables considered during the stepwise process but not included in the final model were gender; stratifying variables at baseline, namely, cohort, suicidality, and current antidepressant use; and moderators at baseline, namely, episodes of major depression, Beck Depression Inventory, Beck Hopelessness Scale, and Beck Scale for Suicide Ideation. ^b^ As the two contrasts of interest (MBCT vs. CPE and MBCT vs. TAU) are not orthogonal, we derived these rows by rerunning the model with group contrasts MBCT vs. TAU and (MBCT or TAU) vs. CPE (not shown).					

**Table 4 tbl4:** Relapse by Treatment Group by Childhood Trauma Questionnaire (CTQ) Scores

Variable	Hazard ratio	CI	*p*
Low CTQ scores^a^			
MBCT vs. CPE	1.17	[0.66, 2.08]	.59
MBCT vs. TAU	1.30	[0.67, 2.51]	.44
CPE vs. TAU	1.13	[0.56, 2.26]	.74
High CTQ scores^b^			
MBCT vs. CPE	0.61	[0.34, 1.09]	.09
MBCT vs. TAU	0.43	[0.22, 0.87]	.01
CPE vs. TAU	0.72	[0.39, 1.32]	.29
*Note*. CI = confidence interval; MBCT = mindfulness-based cognitive therapy; CPE = cognitive psychological education; TAU = treatment as usual.			
^a^ Low CTQ scores: MBCT *n* = 55, CPE *n* = 44, TAU *n* = 30. ^b^ High CTQ scores: MBCT *n* = 44, CPE *n* = 59, TAU *n* = 23.			

## Footnotes

1 When the participant gave a month of onset, this was approximated to the 15th of the month. Where the participant stated that their episode began at the start of the month (e.g., “beginning of February”), onset was approximated to the 1st of the month. “Early” in the month was approximated to the 8th, and “late” was approximated to the 22nd of the month. “End” of the month was approximated to the final day of the month. Where these algorithms derived a duration of relapse of less than 2 weeks (e.g., the participant stated that the episode both started and ended in “January” with no further details, leading to initial start and end dates of January 15), the dates were expanded equally in both directions to produce a 2-week relapse period (e.g., 8th–22nd) because the research assessor had always ensured that the symptoms had been present for at least 2 weeks.
